# Vaccine a promising immunotherapy option for head and neck cancer patients

**DOI:** 10.12669/pjms.40.7.8791

**Published:** 2024-08

**Authors:** Syed Fareed Mohsin

**Affiliations:** 1 Syed Fareed Mohsin Associate Professor, Department of Oral and Maxillofacial Diagnostic Sciences. College of Dentistry, Qassim University, Buraydah, Saudi Arabia

**Keywords:** Checkpoint receptors, Head and neck cancer, Immunotherapy, Therapeutic Vaccine

## Abstract

Head and neck cancer (HNC) is a diversified group of tumors arising from the upper aerodigestive tract, encompassing the oral cavity, larynx, and pharynx. Globally, this particular cancer ranks sixth in prevalence, resulting in an annual mortality rate above 325,000 individuals. Surgery, radiation, and chemotherapy are the primary therapeutic options for HNC, which are frequently used in combination. Despite their extensive use, these treatments are typically unsuccessful and can significantly impair patient quality of life. Therapeutic vaccinations are administered to cancer patients instead of preventative immunizations administered to a healthy population. The efficacy of this modality has considerably transformed the application and success of cancer management by providing an additional and effective therapeutic option for patients. Cancer treatment has been revolutionized by introducing Immune Checkpoint receptors inhibitors (ICR), such as anti-CTLA4, anti-PD-1, and anti-PD-L1.3. ICR have also established immunity against self-generated cancerous cells. Cancer vaccines have shown extraordinary synergistic potential with checkpoint inhibitors to maximize tumor-specific CD8+ expansion and activity, which detects and destroys tumor cells. Personalized neoantigen vaccination therapies can potentially combat the heterogeneity of each patient’s tumor. The findings of this review suggest that recent advances in cancer immunology and genetics imply that cancer vaccination can be a promising alternative treatment for head and neck cancer patients. This review conducted a comprehensive literature search to identify relevant studies on immunotherapy options for head and neck cancer patients. The search strategy was designed to capture a wide range of peer-reviewed articles, conference proceedings, and grey literature from 2013 to 2023. The databases searched to ensure comprehensive coverage of the literature included PubMed, Web of Science, and Google Scholar; to include grey literature and articles not indexed in traditional databases.

## INTRODUCTION

Head and neck cancer (HNC) is the seventh most common cancer globally, accounting for more than 660,000 new cases and 325,000 deaths each year.^1^,^2^ It appears that the incidence of this disease has increased in developing nations as it is one of the most common cancers in these regions. Despite the decline in smoking, particularly in developed countries, potential changes in etiology have been suggested.^3^,^4^

The consumption of cigarettes and alcohol are the primary cause of HNC. 5 It is believed that using smokeless tobacco and paan leads to oral squamous cell carcinoma (OSCC) and oral pre-malignancies in Southern and South-Central Asia.^6^ Areca nut is another well-known carcinogen found in betel quid; it is a combination of areca nut, tobacco, slaked lime and catechu, creating a potent cancer-causing agent.^7^

Human papillomavirus infection is a subset of oropharyngeal cancers; these cancers have shown a rise in cases of head and neck cancer regardless of other carcinogens; it has a global prevalence of 24.9%.^8^

Surgery, radiation, and chemotherapy are the primary therapeutic options for HNC, commonly used in combination. HNC is typically treated with surgery combined with chemoradiation, often resulting in significant morbidity.^9^ Despite their extensive use, these treatments are generally unsuccessful and can significantly impair a patient’s quality of life. In recent decades, advances in our understanding of the underlying biology of HNC have resulted in research exploring novel, more targeted treatment approaches with fewer side effects and causing less substantial harm than current therapy. The emphasis is now on generating anti-tumor immune responses via cancer vaccines.^10^,^11^

The method of directing the immune system to find and destroy cancer cells is known as cancer immunotherapy. The success of immunotherapy for cancer has profoundly changed the cancer treatment rationale, resulting in a fourth pillar of care for cancer patients. Immunotherapeutic methods specifically target cancer cells using elements of the patient’s immune system, minimizing many of the adverse effects of conventional therapy techniques. Various immunotherapeutic agents are available to treat cancer (Table-I).

**Table-I:** Modalities Included in Cancer Immunotherapy.

Extensive range of immunotherapeutic techniques		

Vaccines	Immune modulator	Adoptive cell therapies
Antibodies	PD-L1	Tumor infiltrating Lymphocytes (TILs)
Tumor-associated antigen	TIM3	
Neoantigens	LAG3	Engineered T-Cells (Car-T)
Oncolytic virus	CTLA4	

An essential approach to cancer prevention is vaccination therapy. This review focuses on the underlying mechanism of cancer vaccination, recent developments in antigen selection, and the current status of cancer vaccination therapy for HNC.

### Molecular Mechanism of Cancer Vaccination

The cancer vaccination aims to increase the number of antigen-specific CD4+ and CD8+ T cells capable of recognizing and eliminating tumor cells. Thus, current approaches focus on improving antigen-specific effector T cells by studying endogenous antigen-specific T cell reactions to immunotherapeutic vaccination.^12^ The active involvement of antigen-presenting cells (APCs) in vaccination therapy induces strong and specific T-cell responses towards the antigen. Antigen-presenting cells (APCs) are a distinct subset of immune cells with the unique capability to present antigens to naive CD4+ and CD8+ T cells, hence facilitating their activation.^13^

The development of cancer vaccines has shown remarkable promise in terms of their ability to synergize with ICR (immune checkpoint receptors) blockade to increase the number of tumor-specific CD8+ CTLs and sustain their function through this approach. APCs can be enhanced to mitigate the immune escape of cancer cells. 14

Vaccines have also been revitalized by the success of ICR inhibitors, which provide unique benefits in improving cancer detection by the body’s immune system. Vaccines can cause significant expansions of lymphocytes specific to antigens, according to the cumulative experience of utilizing vaccines to treat infectious illnesses.^15^

The use of vaccines in the context of a “minimal disease” setting is highly appealing in preventing relapse and facilitating recovery after surgical removal of tumors. Due to the immunosuppressive nature of TME (tumor microenvironment), vaccine-induced effector T cells may be rapidly exhausted in advanced-stage tumors. In such circumstances, a combination of a tumor-specific vaccine and an ICR blockade is recommended to achieve long-term tumor regression.

The primary cause of death associated with HNC is the diagnosis of recurrence and metastasis. In 2016, Pembrolizumab (commercially known as Keytruda and manufactured by Merck) and nivolumab (commercially known as Opdivo and manufactured by Bristol-Myers Squibb) have received regulatory approval by Food and Drug Administration as ICR blockers for the treatment of advanced HNC by restoring the function of exhausted cytotoxic T cells. 15% of patients with recurrent or metastatic HNC responded to cetuximab and ICR inhibitors.^12^,^13^ Hence, it would be advantageous to have strategies for predicting anti-programmed death (PD)-1 therapeutic response and resistance. Moreover, predictive tools and novel immunomodulatory medication combinations may enhance the efficiency of cancer therapies.

### HNC Vaccines Design

ICR inhibitors’ success has aroused interest in cancer vaccines, which have a distinct advantage in improving immune detection. Vaccines have been shown to cause robust antigen-specific T-cell expansion in infectious diseases. A vaccine offers great promise in a setting where recurrences are minimal since it promotes healing and prevents recurrences. Advanced tumors may rapidly exhaust vaccine-induced effector T cells due to the immunosuppressive TME (tumor microenvironment). In such circumstances, it is necessary to combine the use of a tumor-specific vaccine with ICR blockade (immune checkpoint receptor). All aspects of cancer vaccine development must be optimized, including antigens, adjuvants, and delivery methods. Vaccination against cancer induces an anticancer CD4+/CD8+ T-cell response specific to cancer antigens. The majority of vaccines are delivered in the form of peptides or DNA or RNA-encoding antigens. 16,17 An effective vaccine peptide should be explicitly expressed within cancer cells, have high immunogenicity and be functionally dependent upon cancer cells. HPV-causing cancers can be treated with the expressed viral antigens, whereas cancers that do not exhibit HPV antigens must be treated using other antigens.

### Target Antigens

There are two distinct categories of cancer antigens, namely and tumor-specific antigens (TSAs) tumor-associated antigens (TAAs). Tumor-specific antigens (TSAs) refer to mutated self-proteins (neoepitopes) that are particular to cancer cells, whereas tumor-associated antigens (TAAs) encompass unmutated self-proteins (such as MUC1 and CEA) or cancer-testis antigens (CTAs)^18^.

### Types of Vaccines

**Fig.1:**
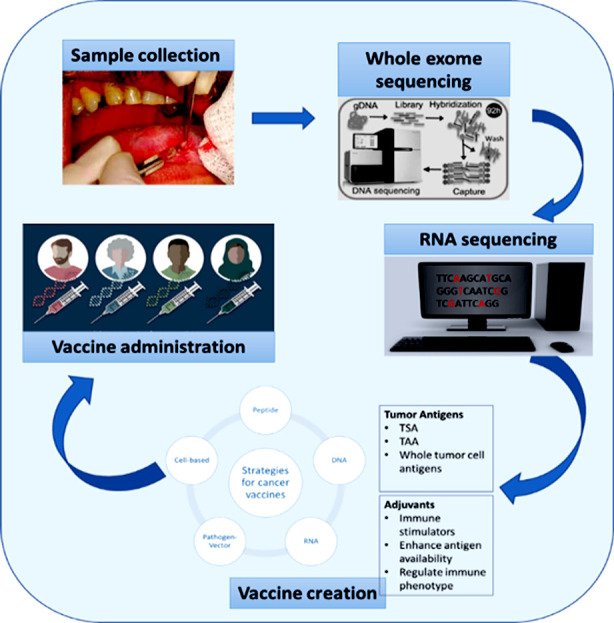
Personalized therapeutic cancer vaccine creation and delivery workflow.

There are five types of HNC vaccines currently being tested in clinical trials: peptide vaccines, DNA vaccines, RNA vaccines, pathogen vector vaccines, and cell-based vaccines (Fig.1). Antigen and adjuvant can be delivered into the same cells to reduce the number of APCs that develop antigen-specific tolerance. Effectively targeting the lymph nodes, minimal side effects, and minimal safety concerns are all desirable characteristics of an effective vaccine. Although each vaccine system has advantages, none can achieve all the above goals.

The most common technology is peptide vaccination, which is considered safe. The amino acid sequences of peptide-based vaccinations contain the epitope that can elicit an immune response. A human leukocyte antigen (HLA) pathway is used to present the injected peptide to naive T lymphocytes after it is picked up by antigen-presenting cells. Subsequently, the activated cytotoxic T lymphocytes will identify the analogous epitope presented on the surface of the neoplastic cells through major histocompatibility complex class I molecules, leading to the eradication of the malignant cells.^19^ DNA and RNA vaccines are more accessible to synthesize than peptide vaccines, making them a better choice for children and adults. Many DNA vaccines contain immunostimulatory molecules like IL-2 and granulocyte/macrophage colony-stimulating factors produced by bacterial plasmids. In addition to their stability, DNA vaccines can activate CD4+ and CD8+ T cells and present to MHC-I and MHC-II by APC.^20^ Pathogen-vector vaccines act as an application of bacteria and viruses as adjuvants in vaccines due to their ability to activate the innate immune system. Cell-based vaccines are developed by expanding tumor antigen peptides ex vivo in the presence of the appropriate cytokines. The use of cell-based vaccines targeting cancer stem cells (CSCs) showed significant improvements in host survival and tumor control in a preclinical model of squamous cell carcinoma.^21^

### Studies of clinical vaccination in HNC

Several clinical studies have been carried out to evaluate the effectiveness of cancer vaccines. As summarized in Table-II, some of those studies have been sourced from ClinicalTrials.gov and PubMed. The HPV antigen-related vaccine has demonstrated its effectiveness and safety. Several others, including TAA, cellular vaccines and neoantigens, have also shown promising results. There are six vaccines against HPV to identify the safety and efficacy with or without other therapies. The rest of the studies focused on carcinomas not associated with HPV infection, Table-II. A study in the National Institutes of Health Clinical Center (NCT04247282) is working to determine if immunotherapy can reduce previously untreated head and neck tumors before surgery or prevent the tumors from returning following complete treatment. One study (NCT02999646) investigated a personalized cell-based, active immunotherapy (MVX-ONCO-1) composed of dead tumor cells from the patient and genetically altered cells to evaluate its effectiveness to increases the immune system’s response to tumor cells by inducing an immunological reaction against the patient’s cancer cells. NCT03568058 has entered Phase-Ib to examine the effectiveness of anti-PD-1 (pembrolizumab) and personalized cancer vaccine in patients with advanced cancer. A Phase-I clinical trial (NCT04266730) that aimed to assess the safety of administering the personalized and adjusted neoantigen peptide vaccine (PANDA-VAC) in combination with pembrolizumab to individuals diagnosed with advanced squamous non-small cell lung cancer (NSCLC) or squamous cell carcinoma of the head and neck. The research findings suggest that immunization is a potentially effective technique for treating HNC. However, additional research is required to confirm the efficacy and safety of therapeutic vaccinations.

**Table-II:** Selected clinical trials for cancer vaccines.

Trial Number	Vaccine ± other therapy	Type of vaccine	Phase	Target antigen	N	Primary endpoint
NCT02002182	ADXS11-001 (ADXS-HPV)	Live (Listeria Monocytogenes)	II	HPV	15	Response and safety
NCT03821272	PepCan	Peptide	I/II	HPV	20	Efficacy and safety
NCT02865135	DPX-E7	Peptide	Ib/ II	HPV-16 E7	11	Safety
NCT03260023	TG4001 + Avelumab	Live (modified vaccinia Ankara virus)	Ib/ II	HPV16 E6/E7	150	I: Safety II: Efficacy
NCT03418480	Hare-40	RNA	I/II	HPV16 E6/E7	44	I: Safety II: Efficacy
NCT04369937	ISA101b + Pembrolizumab + Cisplatin + radiotherapy	Peptide	II	HPV16 E6/E7	50	Efficacy
NCT02544880	Anti-MUC1 Vaccine + Tadalafil	Peptide	I/II	MUC1	16	Efficacy
NCT04247282	M7824 + N803+ TriAd vaccine	Live (Adenovirus)	I/II	Brachyury, Mucin-1, and CEA	21	Efficacy
NCT02999646	MVX-ONCO-1	Cell-based and Personalized	II	Autologous tumor cells	21	Safety
NCT03568058	Pembrolizumab + neoantigen	Personalized	I	Tumor-derived neoantigen	30	Safety
NCT04266730	PANDA-VAC + Pembrolizumab	Personalized peptide	I	Neoantigen	6	Safety

### Combined Cancer Therapy Considerations

Numerous methodologies to strengthen the immune system’s ability to combat cancer by generating a subset T-lymphocytes (CD8+) that can selectively recognize tumor antigens and destroy tumor cells. Vaccine-based therapies often exhibit limited efficacy as standalone treatments due to the immunosuppressive conditions within tumors, which impede the activation of vaccine-induced T cells. There are several mechanisms that can contribute to immune tumor escape, including the activation of immune checkpoint inhibitors such as PD-1/PD-L1 and CTLA4, as well as molecules like mucin domain-containing molecule (TIM)3, T cell immunoglobulin and lymphocyte activation gene (LAG)3. Additionally, immune tumor escape can be facilitated by extrinsic pathways involving regulatory T cells (T-regs) or myeloid-derived suppressor cells, as well as the release of cytokines like transforming growth factor-beta (TGF-β).^22^ Thus, therapies that affect various immune responses to cancer stages may help to increase immunotherapy’s effectiveness.

A cancer vaccination may increase antigen-specific T cells, enhancing the effect of CPIs.^14^ The inhibitors of the EZH2 enzyme repressed H3K27me3 epigenetically enhance antigen presentation. These findings suggest combining EZH2 with cancer vaccination may be a promising approach.

### Vaccines in Neoadjuvant Immunotherapy

Since surgery alone for locally advanced HNC traditionally had poor outcomes, postoperative adjuvant therapies for locally advanced HNC have been researched for many years. Nonetheless, the five-year survival rate for advanced HPV-negative HNC patients is still under 50%.^23^ Neoadjuvant therapy is used as the first line of treatment to boost immunity against tumors to improve clinical outcomes. Neoadjuvant immunotherapy differs from other forms of immunotherapy since it is offered to treat naïve tumors and thus lacks treatment-resistant cells; moreover, it uses the host immune system to attack tumor cells persistently. Several studies have demonstrated encouraging results when a therapeutic vaccine was given before surgery.^24^ Neoadjuvant checkpoint inhibitors demonstrated improved survival relative to the adjuvant setting in a spontaneous mouse metastatic breast cancer model by reducing metastatic lesions.^25^ CPIs in the neoadjuvant setting provided enhanced systemic T-cell responses to tumor-specific antigens before surgery.^26^ A study by Schoenfeld et al. (NCT02919683) advocated the effectiveness and reduced undesirable effects of neoadjuvant therapy in oral cancer patients.^27^ A Phase-II trial (NCT02296684) with pembrolizumab supported the therapy’s safety and showed a marked reduction in the one-year relapse rate.^28^ Neoadjuvant immunotherapy has been demonstrated to be safe and does not delay surgical modality.^27^,^28^ The studies mentioned investigated neoadjuvant therapy prior to surgery; however, this approach is also being explored with radiation and chemotherapy with promising results.^29^,^30^

### Limitations and Future Directions

Several patients receiving immunomodulatory therapy, including CTLA-4, PD-1, or its ligand PD-L1, have little to no benefit at the expense of significant harm. Therefore, it is necessary to identify the molecular factors determining how well immunotherapy works.

Production costs and patient access are two of the biggest obstacles to vaccinations, particularly personalized vaccines.^31^ Whole exome sequencing and bioinformatics predictions are required for personalized vaccines (Fig.1) and are presently undergoing trials. However, the availability of these vaccinations is currently limited for patients with advanced HNC who require quick and effective treatment therapy. Recurrent or metastatic HPV-negative HNC patients have a short life expectancy, logistical support, time, and cost constraints exist. However, HNC vaccines promise to prevent cancer recurrence and reduce treatment for HPV-positive HNC patients. Many new tumor-specific CTLs (Cytotoxic T lymphocytes) will be exhausted in the TME (tumor microenvironment).

The use of red blood cells (RBCs) for autologous blood cell-based vaccination is another intriguing alternative strategy now being researched. As RBCs can be engineered to primarily interact with the spleen and blood vessels, this strategy avoids the side effects linked to other immunomodulatory proteins that spread to all organs.^32^

In addition to cost-effectiveness, vaccines with good safety profiles are highly desired. No vaccine delivery method has successfully attained these objectives. One illustration is that, cell-based vaccines are immunogenic, but their production is costly, labor intensive, and lacks standardization. Patients with immunocompromised conditions, such as HIV or those receiving myelosuppressive chemotherapy, are at risk from pathogen vector vaccines. Nanoparticles are highly promising for delivering vaccines because they safeguard vaccine constituents against degradation and possess excellent lymph node-homing properties. However, many nano-vaccines are using to induce anti-tumor immune responses in mice. Because human APCs lack TLR9 expression, more testing is required. It is essential to optimize the delivery of CD4+ and CD8 T-cell epitope ratios and the combinatorial protocol to maximize the potential of cancer vaccines.

## CONCLUSION

Recent breakthroughs in cancer immunology and genomics suggest that cancer vaccination can be a personalized and highly efficacious treatment method. HNCs have a high TMB (tumor mutational burden) making cancer vaccination therapy a useful treatment option for treatment-resistant HNCs. Notably, recent advances in NGS (next-generation sequencing) and MS (mass spectrometry) have improved the detection of strong neoepitopes. The development of cancer vaccines and the analysis of the results from the clinical trials will aid in advancing this modality.

Developing personalized neoantigen vaccine treatments may effectively address tumor heterogeneity in each patient. Neoepitope detection and vaccine delivery remain challenges. Researchers are addressing these issues at a rapid pace in this field and will potentially alter how HNC is treated in the future. Overall, personalized cancer vaccines are the future of HNC immunotherapy. It is expected that the incidence of HPV-positive HNC will increase until 2060. By developing improved HPV oncoprotein-targeted vaccine delivery systems, morbidity can be reduced, and cure rates will be improved. The use of cancer vaccines, combined with ICR-targeted treatments, will likely lead to a long-lasting, robust anti-HNC immune response.

The available evidence suggests that these vaccines can be used as adjuvants to other established regimens for the treatment of HNC, such as CPI or chemoradiation. Further preclinical and clinical research is required to validate the additional functions of these therapeutic vaccinations.

### Abbreviations

HNC, Head and neck cancer; APC, antigen-presenting cell; cDC1, conventional type 1 dendritic cell; cDC2, conventional type 2 dendritic cell; CPI, immune checkpoint inhibitor; CR, complete response; CRT, chemoradiotherapy; CSCs, cancer stem cells; CTA, cancer testis antigen; DC, dendritic cell; EBNA1, Epstein-Barr nuclear antigen 1; EZH2, enhancer of zeste homolog 2; HLA, human leukocyte antigen; HNC, head and neck squamous cell carcinoma; HPV, human papillomavirus; ICRs, immune checkpoint receptors; IFN-γ, interferon gamma; IL, interleukin; indel, insertion/deletion; LMP2, latent membrane protein 2; LN, lymph node; MS, mass spectrometry; NGS, next-generation sequencing; OS, overall survival; PD-1, programmed cell death-1; PD-L1, PD-1 ligand; PFS, progression-free survival; Rag, recombination activating gene; RNA-seq, RNA sequencing; SLP, synthetic long peptide; SNV, single nucleotide variant; TAA, tumor-associated antigen; TAP, transporter associated with antigen processing; TCR, T-cell receptor; Th1, type 1 helper T cell; TMB, tumor mutational burden; TSA, tumor-specific antigen; TLR9, Toll cell receptor9.
